# 

**Published:** 1886-06

**Authors:** 


TO NEW SUBSCRIBERS.

All new subscriptions for Vol. VII. received after this, must commence with the July number. A sufficient number of extra copies was printed, it was thought, to supply all the new subscribers who would wish to commence with the beginning of the volume. Yet every one has been used, and a considerable number of subscriptions sent in, with a request for back numbers, have been necessarily entered as beginning with the July number. The demand has been much greater than our anticipations. If any one has January and February numbers which he does not desire to keep, and will send them to the Buffalo office, it will enable us to supply some who earnestly desire to commence with the volume.

